# Numerical Simulation of Dynamic Degradation and Fatigue Damage of Degradable Zinc Alloy Stents

**DOI:** 10.3390/jfb14110547

**Published:** 2023-11-15

**Authors:** Jing Qi, Hanbing Zhang, Shiliang Chen, Tianming Du, Yanping Zhang, Aike Qiao

**Affiliations:** Faculty of Environment and Life, Beijing University of Technology, Beijing 100124, China

**Keywords:** degradable zinc alloy stents, fatigue damage, pulsating cyclic loading, dynamic degradation

## Abstract

Current research on the fatigue properties of degradable zinc alloy stents has not yet considered the issue of the fatigue life changing with material properties during the dynamic degradation process. Therefore, in this paper, we established a fatigue damage algorithm to study the fatigue problem affected by the changing of material properties during the dynamic degradation process of the stent under the action of pulsating cyclic loading. Three models: the dynamic degradation model, the dynamic degradation model under pulsating cyclic loading, and the coupled model of fatigue damage and dynamic degradation, were developed to verify the effect of fatigue damage on stent life. The results show that fatigue damage leads to a deeper degree of inhomogeneous degradation of the stent, which affects the service life of the stent. Fatigue damage is a factor that cannot be ignored. Therefore, when studying the mechanical properties and lifetime of degradable stents, incorporating fatigue damage into the study can help more accurately assess the lifetime of the stents.

## 1. Introduction

Coronary artery stenosis is currently a relatively serious cardiovascular disease. Percutaneous coronary intervention (PCI) has become the main treatment method for such diseases [1]. PCI mainly applies various materials of stents to implant diseased blood vessels, including permanent and biodegradable stents. To avoid the possibility of secondary injuries to patients and to reduce the difficulty of reoperation by doctors, biodegradable stents have been widely researched. Zinc alloy as a biodegradable material can completely degrade in vivo, reducing the need for secondary surgical removal. Compared with magnesium alloys with stronger corrosion resistance, the corrosion rate of zinc alloys is closer to the remodeling rate of human tissues and blood vessels. Therefore, zinc alloy materials are widely studied in the medical field such as cardiovascular stents and orthopedic implants [2,3,4,5,6,7,8].

However, the stent fracture is a common problem after implantation [9,10,11], especially for biodegradable stents. The stent fracture is mainly due to the fatigue of the stent itself. The probability of stent fracture after stent implantation ranges from 3.7% to 7.7% [12]. Stent fracture increases the probability of thrombus formation within the stent, thereby increasing the fatal risk for patients.

For degradable stents, which gradually become thinner and thinner under the corrosive effect of human blood ions. The corrosive behavior of degradable stents mainly includes uniform corrosion, stress corrosion, and localized pitting corrosion. Under the influence of the above corrosive effects, the degradable stent gradually degrades with changes in the material properties and effective stresses.

The stent is always subjected to pulsating cyclic loading. Meanwhile, degradable stents also accompanied the changes in material properties and ion environments caused by the degradation. Therefore, the service environment of biodegradable zinc alloy stents is more complex, and the mechanical properties are also more difficult to predict. According to the physiological environment, the fracture of degradable stents is mainly caused by fatigue corrosion. The principle of fatigue corrosion is a fracture process of a stent under corrosive environments and alternating loading. In the case of PCI, the stent is subjected to the erosive of blood ions and the repeated loads of pulsating cyclic loading. When the stent undergoes corrosion, depressions such as pits or cracks will appear on its surface. These depressions are prone to stress concentration during repeated loading, accelerating the expansion of the pits and cracks of stents. The above phenomena can cause more severe damage to the stent and eventually cause the stent to fracture [13].

Previous studies showed that the corrosive effect of a single blood ion or the effect of a single pulsating cyclic loading can affect the mechanical properties of the stent. On the other hand, the pulsating cyclic loading accelerates the progress of corrosion damage, and corrosion damage promotes the evolution of damage [14,15,16]. Considering the real vascular environment, it is necessary to include the fatigue damage caused by pulsating cyclic loading in the corrosive state in the evaluation of stent mechanical properties. This can reflect the performance changes of the stent in a more comprehensive and accurate way. The existing studies on corrosion fatigue related issues do not consider the issue of material property changes during the degradation of the stent [14]. Due to the change in material properties, the fatigue life of the material will also change, which will affect the fatigue life of the stent. It is necessary to consider the change of material properties during degradation as well as fatigue damage.

For fatigue damage under a corrosive state, scholars have studied the corrosion fatigue mechanism of a variety of metal materials [17,18,19]. Most scholars used in vitro experiments to study the fatigue properties of metal materials by applying cyclic loading to them in simulated body fluids. In vitro experiments generally take several months, consume a lot of resources, and are affected by many unstable factors due to the environment [20,21]. More and more scholars studied the mechanical properties of degradable stents from the perspective of finite element simulation. Numerical simulation is more intuitive for experiments and easier for data processing. The loading required for the material can be in any direction, which cannot be achieved by in vitro experiments. Therefore, numerical simulation methods are now widely used for material characterization, such as simulating and observing cracks [22,23,24,25,26].

Based on the principle of continuous damage mechanics (CDM), a suitable model of the degradable zinc alloy stent was established in this paper. During the degradation process of the stent, the material properties gradually change with the effect of corrosion. The fatigue life of the stent may be affected due to the change in material properties. This is ultimately due to the S-N curve, where the force changes and the fatigue life also changes. In this paper, the fatigue life was incorporated into the damage algorithm through the functional relationship of S-N, and the fatigue damage caused by different stresses was accumulated through the Miner linear accumulation criterion. Then fatigue damage and corrosion damage were coupled to study the mechanical property changes of degradable zinc alloy stents during the dynamic degradation process.

## 2. Materials and Methods

### 2.1. Model Construction and Setting of Material Properties

**Figure 1:**
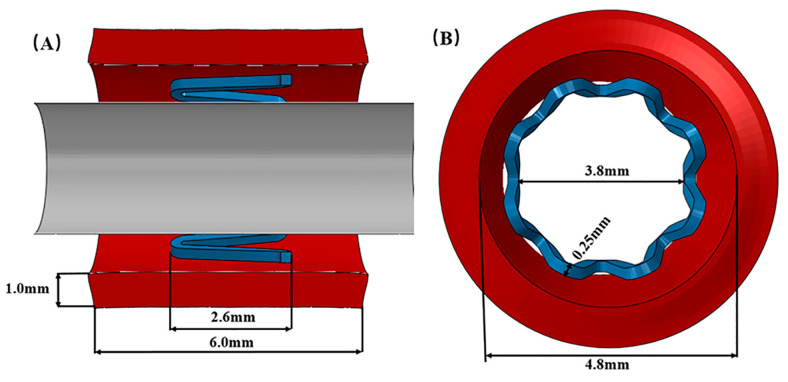
(**A**), Longitudinal section of the stent; (**B**), Cross-sectional view of the stent.

In this paper, the stent model was simplified, and a single-ring stent was designed for a numerical simulation study [27] (Figure 1). The model was built using SolidWorks 2016 (SolidWorks Inc., Waltham, MA, USA). The material of the stent was defined as a zinc alloy material (Table 1); the blood vessel was defined as a hyperelastic material by using the third-order Ogden’s eigenequation [28], as shown in Equation (1). Both the blood vessel and the stent were modeled as homogeneous, isotropic elastic-plastic materials; and the balloon was defined as a shell element. All element types were set to linear reduced integration elements C3D8R, this process was accomplished through the Hypermesh 14.0 (Altair Engineering Inc., East Big Beaver Road Troy, MI, USA).

(1) $$W=\sum_{i=1}^{N}\frac{\mu_{i}}{\alpha_{i}}({\bar{\lambda_{1}}}^{\alpha_{i}}+{\bar{\lambda_{2}}}^{\alpha_{i}}+{\bar{\lambda_{3}}}^{\alpha_{i}}-3)+\sum_{k=1}^{N}\frac{1}{D_{k}}{\left(J-1\right)}^{2k}$$

W is a strain energy density functional, J is an elastic volume ratio, $\lambda_{1},{\;\lambda }_{2},\lambda_{3}$ is the main elongation, $D_{k}$ is a coefficient that describes the compression properties of a material, $\mu_{i}$ and $\alpha_{i}$ relate to the frontal shear behavior of the material. Detailed parameters are shown in Table 2.

### 2.2. Stent Expansion-Recoil and Pulsatile Compression Loading

The stent implantation process was simulated using the Abaqus 6.14/Standard solver (DS-SIMULIA, Providence, RI, USA), and simplified into two stages: expansion and recoil.

The stent and vessel are in face-to-face contact (friction factor 0.2). Circumferential restraints were applied to both ends of the balloon to prevent any rotation of the balloon during expansion. At the same time, circumferential and axial constraints were applied to both ends of the vessel to prevent arbitrary rotation of the vessel. A radial displacement loading was applied to the balloon to simulate the process of the stent supporting the vessel, expanding the stent to 1.1 times the size of a normal healthy vessel. After the stent was fully expanded, the expansion loading was unloaded, and the vessel and the stent underwent elastic recoil [29].

**Figure 2:**
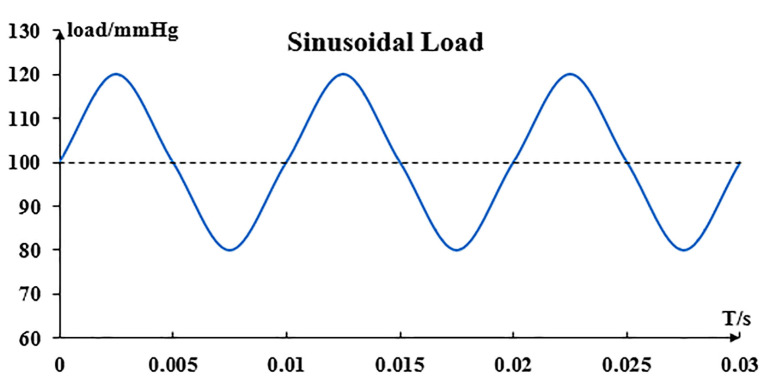
Pulsating cyclic loading.

The biodegradable zinc alloy stent implanted in the body is subjected to stresses, including residual stress from stent deployment and pulsating cyclic loading from cardiac ejection. This paper considers that the stent has already been subjected to pulsatile pressure during the implantation process, so a basic pressure of 100 mmHg is applied to the vascular wall during the expansion. Then this paper considers the effects of pulsating cyclic loading to which the stent is subjected during service. A cyclic loading of ±20 mmHg is applied to the vessel to simulate a pulsating cyclic loading of 80–120 mmHg applied to the stent (Figure 2). For the problem of cyclic loading, the focus of this paper is to provide a cyclic loading condition for the blood vessels, and the average stress correction problem is not considered for the time being.

### 2.3. Corrosion Fatigue Algorithm Model

The CDM theory focuses on the effect of damage on the macroscopic mechanical properties of materials. This paper focused on the process and pattern of the evolution of material properties and structural damage of degradable zinc alloy stents over time. The evolution of damage during fine-scale physico-mechanical processes in the material is not considered.

The macroscopic mechanical behavior of the material during damage is described realistically by the internal damage variable D. The effect of damage on the macroscopic mechanical properties of the material and the damage evolution process of the structure are investigated.

The formula for calculating the effective stress in the metallic material during damage is shown below [15]:

(2) $$\sigma =\bar{\sigma }\left(1-D\right)$$

where:σ—effective stress; $\bar{\sigma }$—stress when undamaged.

*D* is the damage field function. *D* = 0 indicates that no damage to the material has occurred, and *D* = 1 indicates that the material is completely damaged.

Due to the gradual corrosion and thinning of degradable stents in the human body, the strength and Young’s modulus of the stent gradually decrease with corrosion. The stress-strain relationship is shown in Equation (3).

(3) $$\varepsilon =\frac{\sigma }{E}=\frac{\bar{\sigma }}{\bar{E}}=\frac{\sigma }{E(1-D)}$$

ε is strain; σ is stress; E is Young’s modulus. A stress corrosion crack is induced by the residual stress accumulated from plastic deformation. It shows that the material properties are dependent on damage variable *D*. According to the principle of strain equivalence assumption, Young’s modulus changes as follows:

(4) $$E=\bar{E}(1-D)$$

where: E—Young’s modulus when damaged; $\bar{E}$—Young’s modulus when undamaged.

Shen [14] et al. developed a fatigue model for magnesium alloys. In their study, the damage of the stent caused by cyclic loading was included in the total damage variable D.

Whereas corrosion damage and fatigue damage are linearly superimposed on the metallic material, the damage field variable *D* is shown in Equation (5):

(5) $$D=D_{U}+D_{SC}+D_{P}+D_{f}$$

*D_U_*—Uniform corrosion damage; a corrosion phenomenon that occurs at a constant rate on the surface of a degradable metal stent.

*D_SC_*—Stress corrosion damage; a process of material destruction resulting from the combination of stress and a corrosive environment.

*D_p_*—Pitting corrosion damage; a corrosion phenomenon that is concentrated on a small area of the metal surface and can penetrate deep into the metal.

*D_f_*—Fatigue damage; the accumulation of damage during cyclic loading of metal structure can lead to crack initiation or expansion, with cracks eventually leading to fracture of the structure.

Uniform corrosion is a type of micro-current corrosion, which begins when the component is exposed to a corrosive environment and in contact with the corrosive medium [15]. Therefore, uniform corrosion first occurs on the surface of the stent and the principle formula is shown in Equation (6).

(6) $$\frac{dD_{U}}{dt}=\frac{\delta_{U}}{L_{e}}k_{U}$$

$\delta_{U}$ is a feature size in uniform corrosion; $L_{e}$ is the characteristic length of the element in finite element analysis; $k_{U}$ is a kinetic parameter related to uniform corrosion, the values of the relevant parameters are in Table 3.

Stress corrosion is mainly related to the stress state of the component, and the main condition for its occurrence is that the equivalent stress $\sigma_{eq}^{*}$ of the component exceeds the stress corrosion threshold $\sigma_{th}$. The principle formula is shown in Equation (7). If the equivalent stress $\sigma_{eq}^{*}<\sigma_{th}$, then ${dD}_{sc}=0$ [15]. For stress corrosion threshold $\sigma_{th}$ is generally considered to be from 30% of the yield stress of metal materials to 90% of the ultimate tensile stress [32].

(7) $$\frac{dD_{SC}}{dt}=\frac{L_{e}}{\delta_{SC}}{\left(\frac{S\sigma_{\mathrm{eq}}^{*}}{1-D_{SC}}\right)}^{R}$$

(8) $$\sigma_{eq}^{*}=\frac{1}{\sqrt{2}}\sqrt{{\left(\sigma_{x}-\sigma_{y}\right)}^{2}+{\left(\sigma_{y}-\sigma_{z}\right)}^{2}+{\left(\sigma_{z}-\sigma_{x}\right)}^{2}+6(\tau_{xy}^{2}+\tau_{yz}^{2}+\tau_{zx}^{2})}$$

The C3D8R elements are reduced-integration elements, each with one integration point and six forces in each direction. Where stresses extracted into the element in six directions are calculated $\sigma_{eq}^{*}$; *S* and *R* are constants related to the dynamics of the stress corrosion process; $\delta_{SC}$ is a characteristic dimension of the stress corrosion process, the values of the relevant parameters are in Table 3.

Pitting corrosion is a localized form of corrosion in which small holes are formed on the surface of a component under the influence of a corrosive medium. The principle equation is shown in Equation (9) [33].

(9) $$\frac{dD_{P}}{dt}=\frac{\delta_{U}}{L_{e}}\lambda_{e}k_{U}$$

(10) $$\lambda_{e}=\beta \lambda_{n}$$

where $\lambda_{e}$ is the pitting corrosion parameter, which is randomly assigned to each element on the initially exposed surface by a random number generator based on the standard Weibull distribution. $\lambda_{n}$ is the pitting parameter of the removed element; *β* is a dimensionless parameter that controls the acceleration of pit growth, the values of the relevant parameters are in Table 3.

In this paper, a fatigue damage accumulation algorithm is established with Miner’s linear accumulation criterion and Basquin’s equation. Fatigue damage factors are introduced on the basis of dynamic degradation.

The principle of fatigue damage focuses on that damage under loading conditions is a cumulative process. Miner linear accumulation criterion [34] is described as a linear approach to calculate the cumulative damage of loading. According to the S-N curve, it is known that the theoretical fatigue life of the stent is different at different stress levels, so the degree of damage suffered is also different. Under the Miner linear accumulation criterion, the damage to the stent increases linearly for a single stress level.

**Figure 3:**
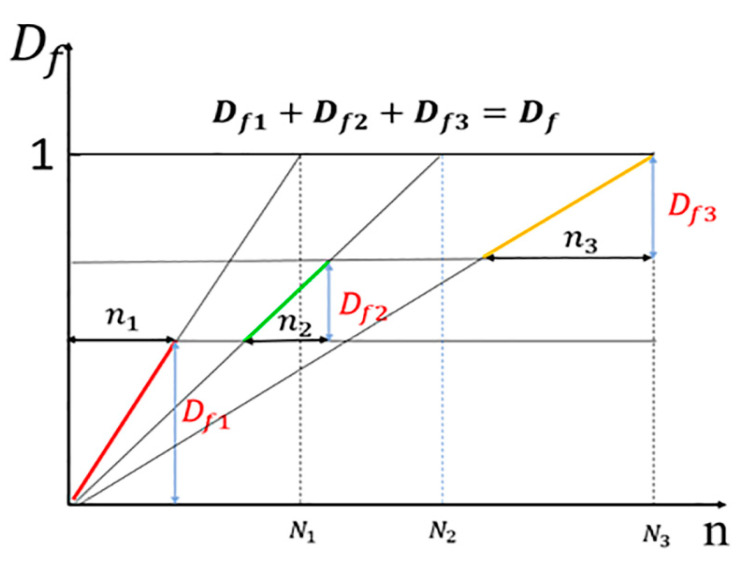
Schematic diagram of Miner linear accumulation theory, the horizontal axis indicates the number of cycles, and the vertical axis indicates the damage value. The damage at different stresses is independent and different colors are used to indicate different damage accumulations. The total fatigue damage is a cumulative process of damage values at different stresses.

The damage to the stent at different stress levels is not simply proportional, but rather the superimposed effect of a number of different levels of damage values. The reason is as follows: Stent damage at different stress levels is not a simple linear trend, different stresses cause different slopes of straight lines of damage to the stent (Figure 3). From the process of degradation, it can be seen that the stress borne by the stent is constantly changing, and then the damage value of the stent also shows a discontinuous linear increase.

Miner linear accumulation theory assumes that the damage caused by one cycle is:

(11) $$D_{f}=\frac{1}{N}$$

where *N* is the fatigue life under the corresponding loading, if the damage caused by *n* cycles under equal amplitude load, it results in:

(12) $$D_{f}=\frac{n}{N}$$

The damage value with time is shown in Equation (12). The selection of fatigue damage stress values is the same as the selection of stress corrosion stress values. where *N_t_* is the number of theoretical loadings at the current fixed stress value obtained from the S-N curve based on Basquin’s equation. According to Basquin’s equation, it can be seen that the theoretical fatigue life of the stent changes with the change of stresses. *n_t_* is the actual cyclic loading, which is related to the frequency *f*.

(13) $$dD_{f}=\frac{n_{t}}{N_{t}}=\frac{fdt}{A\sigma^{-k}}$$

**Figure 4:**
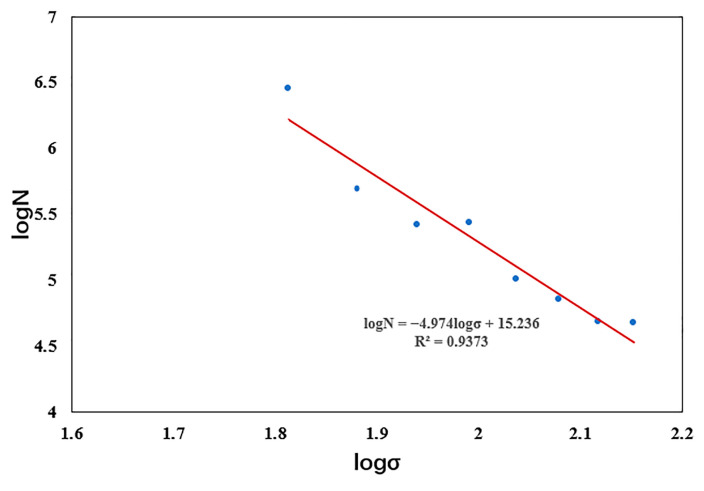
log N versus log σ curves of the zinc alloys. The curve reflects the relationship between stress and fatigue life, with the horizontal axis indicating stress and the vertical axis indicating the number of cycles.

This paper lacks the relevant fatigue test to obtain the S-N theoretical graph of the degradable zinc alloy material. So the relevant data given in the literature [35] were used for a numerical simulation study (Figure 4).

The fatigue loss values *dD_f_* at different stresses are superimposed to be the total cumulative fatigue damage suffered by the biodegradable stent during cyclic loading, as shown in Equation (14).

(14) $$D_{f}=\sum_{t=1}^{i}\frac{n_{t}}{N_{t}}$$

**Figure 5:**
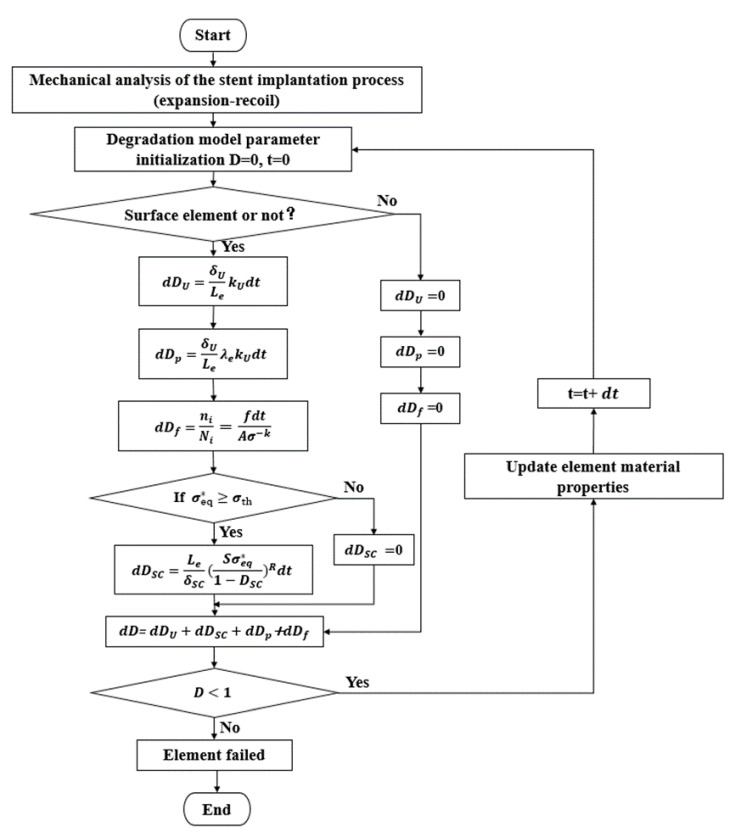
Flow chart of the algorithm.

By writing the corresponding user subroutine (Figure 5), the gradual degradation process of degradable stents under the effect of three kinds of corrosion damage and one kind of fatigue damage is simulated.

In this paper, the residual stress field after stent expansion and recoil was used as the initial stress field for stent corrosion degradation, and pulsating cyclic loading was applied to the intima-vascular wall. According to the definition of fatigue, the stent is prone to fatigue fracture, because the cyclic loading is repeatedly applied to the surface depression of the stent. Thus, the outer surface of the stent is distinguished by a subroutine that identifies the exposed elements on the surface of the stent. Specify that fatigue damage caused by uniform corrosion, pitting, and cyclic loading begins at the surface of the stent and progressively penetrates deeper into the stent as it degrades [36,37,38]. For stress corrosion, as long as the stress in the element is higher than the stress threshold, it can cause stress corrosion to occur in the element where it is located.

**Figure 6:**
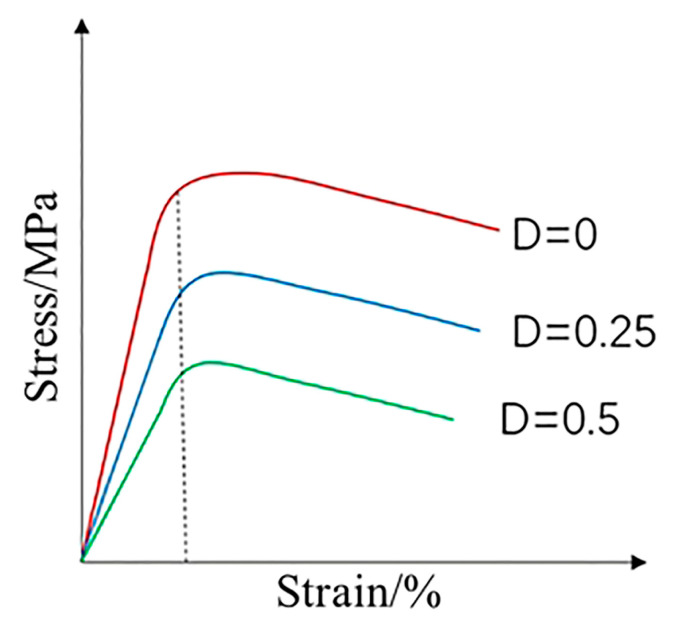
Schematic of linear decrease of material properties.

With the iteration of the time step, the damage value *D* of the stent element starts to accumulate with time. So the material properties and effective stress of the stent show a linear decrease change through the damage value *D* (Figure 6). This process is used to simulate a gradual decrease in the mechanical integrity of the stent element with degradation. When the *D* value accumulates to 1 the corresponding element of the stent is deleted from the stent, completing the simulation of degradation of the degradable stent under pulsating cyclic loading conditions.

### 2.4. Grouping of Corrosion Fatigue Model

The main purpose of this study is to verify the impact of fatigue damage caused by pulsating cyclic loading on the stent, so dynamic degradation and fatigue damage of the stent were modeled. To verify the validity of the model, three sets of experiments were set up to simulate the damage caused by pulsating cyclic loading. The specific experimental grouping is designed as follows:

Control group 1 (C1) is a numerical simulation group of corrosion damage considering uniform corrosion, stress corrosion, and pitting corrosion, for the degradation of group C1 the initial stress condition is the residual stress field after the expansion and recoil of the stent; Experimental group 1 (E1) is a numerical simulation group adding pulsating cyclic loading on the base of C1 to demonstrate the effect of pulsating cyclic loading on corrosion damage. Experimental group 2 (E2) is a numerical simulation group that converts pulsating cyclic loading into fatigue damage on the base of E1. It considered the issue of changes in material properties during dynamic degradation and integrated it into the fatigue damage equation to reflect different degrees of fatigue damage. By comparing the results of E1 and E2, the synergistic effect of corrosion damage and fatigue damage of the stent during dynamic degradation was derived.

## 3. Results

### 3.1. Stress Analysis of the Stent

**Figure 7:**
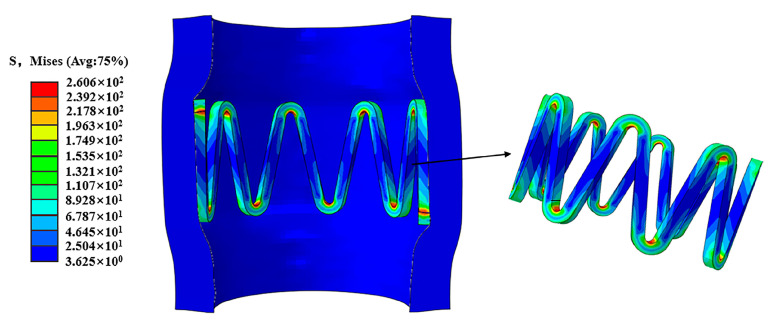
Stress distribution of the stent after expansion and recoil.

The stent plays a certain supportive role in the vessel wall after expansion and recoil. The high-stress region of the stent is mainly concentrated at the corolla of the stent, which is closely related to the degradation morphology of the stent (Figure 7).

**Figure 8:**
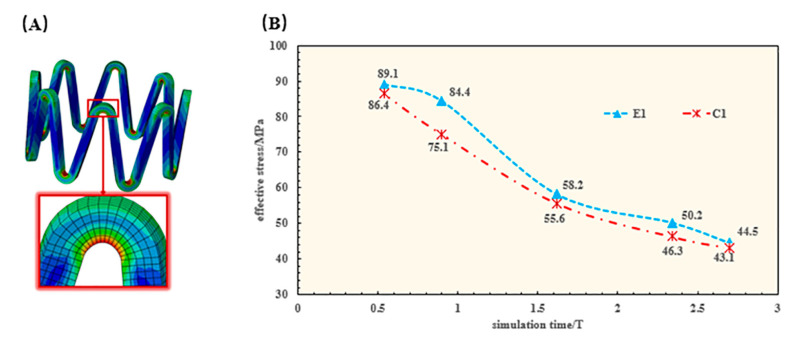
Effective stress of the stent. (**A**), Demonstration of the high stress region of the stent; (**B**), Comparison of effective stresses in the same region of the stent.

The same pulsating cyclic loading conditions were used for E2 and E1, so the effective stresses in the stents were the same. Therefore, the effective stress of the stent is mainly compared between the stent C1 and E1 in the stent of the high-stress region of the effective stress of the element. The trend of effective stress with simulation time T is obtained by calculating the average value of the region (Figure 8).

Because the damage value D discounts the effective stress of the stents, the effective stresses of the stents all show a downward linear decrease as the stents degrade. However, due to the effect of pulsating cyclic loading, the stents are subjected to higher stresses, making the effective stresses of E1 always higher than those of C1 (Figure 8).

**Figure 9:**
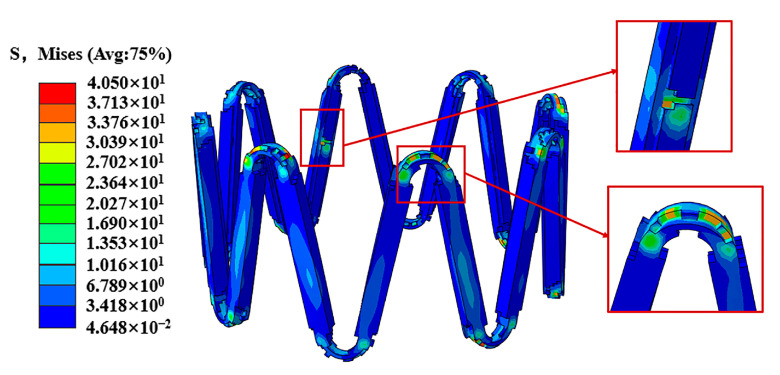
Stress concentration diagram of the stent.

The stress corrosion and pitting corrosion of the stent lead to the existence of pits and other defects on the surface of the stent. The repeated action of pulsating cyclic loading leads to the phenomenon of stress concentration on these defective parts. The stress concentration phenomenon further accelerates the degradation of these areas, leading to premature fracture of the stent and shortening its service life (Figure 9).

### 3.2. Contour of the Damage Field of the Stent

**Figure 10:**
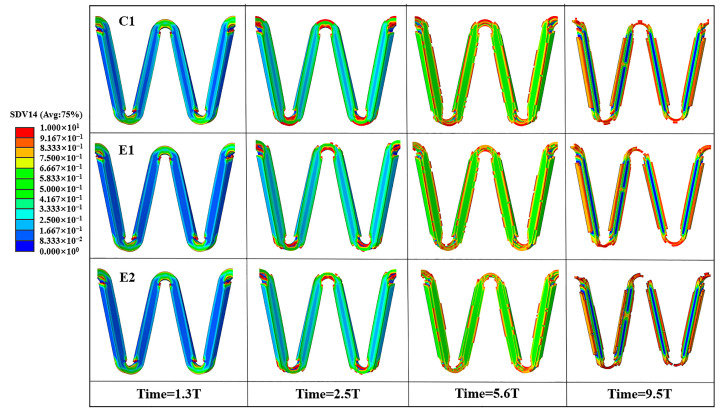
Variation of the damage field of the stent.

**Figure 11:**
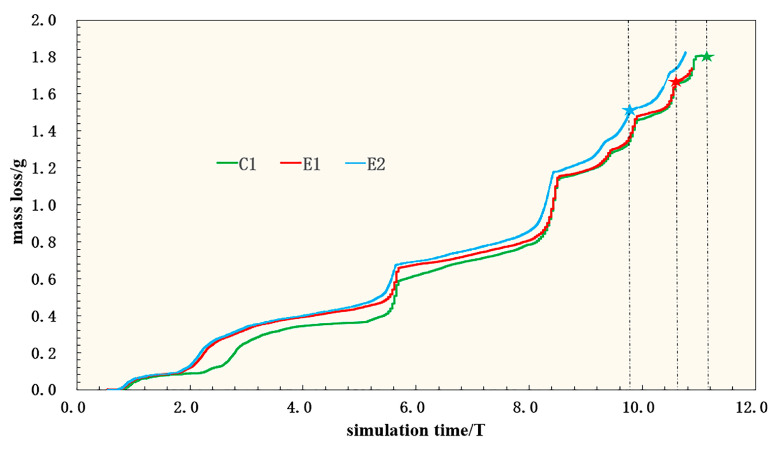
Mass loss diagram of the stent (★ is the point at which the stent breaks).

The total damage force contour of C1, E1, and E2 show that the stents were firstly de-graded in the high-stress region, and E1 and E2 have greater damage and significantly faster degradation (Figure 10). Comparing E1 and C1, the value of stent loss in the high-stress region is slightly larger than the value of damage at the stent rods, so both sets of stents degrade firstly in the high-stress region. Due to the fracture of E1 before C1, it indicates that pulsating cyclic loading can accelerate the degradation process of the stent. From the mass loss diagram (Figure 11), it can be found that the degradation rate of E1 is faster than that of C1 in the early stage; this is due to the fact that the early stent degradation occurs at the corolla in the high-stress region, which is dominated by stress corrosion. The later stage is mainly the thinning of the stent rod, which is dominated by uniform corrosion. The mass loss in the later stage is not very different in the two groups of experiments. However, according to the evaluation of the overall effect, the stent degrades faster due to the effect of pulsating cyclic loading, and fracture occurs firstly in the high-stress region, ending the service process of the stent.

A comparison of the stent damage results for E1 and E2 reveals that the overall degradation of the stents is accelerated after converting the pulsating cyclic loading into the fatigue corrosion damage of the stents (Figure 10). Combined with the mass loss diagram (Figure 11), the damage caused by the pulsating cyclic loading to the stent gradually accumulates, causing the stent to degrade at a later stage of degradation. The degradation speed of the model of E2 is significantly faster than that of the model of E1, which is mainly manifested in the high-stress region. The stent rods are both significantly accelerated, which shortens the service life of the stent.

## 4. Discussion

Cardiovascular stents have been widely studied in the field of biomedical engineering. In this paper, a dynamic degradation model of degradable zinc alloy stents was established based on the principle of CDM. Considering the fatigue life decrease in the degradation process of degradable stents, the effects of blood erosion and cyclic loading on the degradation of zinc alloy stents were simulated by an algorithmic program. The simulation results from the composite model showed that there was a synergistic effect between corrosion damage and fatigue damage, which could lead to faster degradation of the stent. This phenomenon is consistent with that described by Hassan et al. [39].

After the elastic recoil of the stent, it exerted a supportive effect on the vessel wall, and in turn, the reaction of the vessel wall made the stent exhibit the high-stress region that is mainly concentrated at its corolla (Figure 7). The effective stress of the stent gradually decreased during the degradation process of the stent. Based on the loading of pulsatile pressure, the results showed that the pulsating cyclic loading affected the stress profile of the stent where the stent under cyclic loading was found to be stressed more. This result was consistent with the study from Cui et al. [27].

In this work, a significant reduction in the integrity of the stent was induced by corrosion. The degradation occurs firstly at the more stressed corolla, which induces changes in the stent structure. Depressions and stress concentrations on the surface of the stent and uneven degradation of the stent are due to a variety of corrosive effects (Figure 9), This work is consistent with Grogan et al. [33]. The fatigue damage model reflects the experimental results through the structural damage and mass loss of the stent. The fatigue factor added in the model accelerated the corrosion process of the stent, which might make the inhomogeneous degradation pattern of the stent more pronounced.

The degradation of the stent is the result of the combined effect of multiple factors. The damage of pulsating cyclic loading mainly originates from the high-stress region by comparing the structural loss diagram. As a result, the stent breaks firstly at the bending point under pulsating cyclic loading, which plays a very important role in the service life of the stent.

Therefore, when studying the service behavior of degradable stents, it is important to consider both the erosive effect of blood on the stent material and the fatigue damage effect. There is a synergistic effect between the two, which can more accurately predict the service time and fatigue life of the stent.

The work in this paper has some limitations. Firstly, for the establishment of the vascular model, the idealized vascular wall model without plaque was considered. Meanwhile, for the loading of pulsatile pressure, related studies have shown [40,41] that the loading frequency and mode of pulsatile pressure can affect the fatigue problem of the stent. This paper focuses on the effect of fatigue damage factors on stent degradation, using pulsating cyclic loading as a condition. Therefore, a simplified approach to pulsating pressure curve was used without considering complex mean stress corrections and other issues. The effects caused by these factors for the time being will be considered in our future work.

## 5. Conclusions

In this paper, a fatigue damage model of the stent was developed based on Basquin’s equation and Miner’s linear accumulation criterion. The material properties and effective stresses of the stent were updated during the degradation simulation process, and then the fatigue damage effects of degradable stents were analyzed based on S-N curves. The model developed for dynamic degradation showed that in addition to the dominant role of corrosion factors in the degradation of the stent, the pulsating cyclic loading also affected the degradation of the stent. The introduced fatigue damage model explains more comprehensively that the fatigue damage factor of the stent is one of the indispensable factors for analyzing the mechanical properties of degradable stents. This study showed that there was a synergistic interaction between corrosion damage and fatigue damage compared with the common corrosion model, which accelerated the degradation process of the stent and shortened the service time of the stent. This work might provide a reference for the structural optimization design and numerical simulation of the service behavior of degradable stents.

## Figures and Tables

**Table 1:** Stent’s mechanical properties [27].

Density	Young’s Modulus	Poisson’s Ratio	Yield Strength	Ultimate Strength
8.5 g/cm^3^	74,500 MPa	0.3	220 MPa	325 MPa

**Table 2:** Material properties of the vessel wall [28].

$\mu_{1}$	$\mu_{2}$	$\mu_{3}$	$\alpha_{1}$	$\alpha_{2}$	$\alpha_{3}$	$D_{1}$	$D_{2}$	$D_{3}$
−1.84	1.12	0.73	21.71	22	21.2	4.11	0	0

**Table 3:** Degradation parameters of the stent [27,30,31].

$\delta_{U}$	$k_{U}$	$\mu_{3}$	$\sigma_{th}$	S	R	*β*
0.1 mm	0.05/h	0.07 mm	66 MPa	0.005 mm^2^$\sqrt{h}$/N	2	0.8

## Data Availability

Data are contained within the article.
